# Hepatosplenic T-Cell Lymphoma in an Immunocompetent Young Male: A Challenging Diagnosis

**DOI:** 10.7759/cureus.8993

**Published:** 2020-07-03

**Authors:** Mohammad Ammad Ud Din, Ronald Sham, Syed Ather Hussain, Joel Shapiro

**Affiliations:** 1 Internal Medicine, Rochester General Hospital, Rochester, USA; 2 Hematology/Oncology, Rochester Regional Health, Rochester, USA; 3 Pathology, Rochester General Hospital, Rochester, USA

**Keywords:** hepatosplenomegaly, malignancy, t-cell lymphoma

## Abstract

Hepatosplenic T-cell lymphoma is a rare but highly aggressive form of T-cell malignancy. As cases are not routinely seen in practice, the malignancy can be confused with other hematologic conditions that have a similar presentation. Here in, we present the challenges faced in diagnosing a 27-year-old-male who initially presented with asymptomatic pancytopenia and then developed massive splenomegaly over the next three months. After an elaborate workup, including a bone marrow biopsy and extensive serological testing, which all turned out to be negative, he eventually underwent a splenectomy with biopsy results confirming hepatosplenic T-cell lymphoma.

## Introduction

Hepatosplenic T-cell lymphoma (HSTCL) is an uncommon subtype of peripheral T-cell lymphoma, which forms less than 1% of all non-Hodgkin's lymphoma [1]. As the presentation and blood work resemble those of other benign and malignant hematologic conditions, the diagnosis requires a high level of suspicion and is confirmed by a combination of clinical findings with histologic and immunophenotypic analysis of the tissue biopsy [2]. The clinical course of HSTCL is highly aggressive with most patients dying within two years of diagnosis because of disease progression despite the initiation of chemotherapy [1]. To place emphasis on the challenges encountered in establishing the diagnosis, here the authors present a case of a young male who was referred to the hematology clinic by his primary care provider for asymptomatic pancytopenia. He later developed massive splenomegaly over the course of the next three months, eventually requiring a splenectomy with biopsy confirming HSTCL.

## Case presentation

A 27-year-old male of Korean descent with a past medical history of diabetes mellitus type 1 (DM1), major depressive disorder and hepatosteatosis from alcoholism presented with gradually worsening asymptomatic pancytopenia. The initial blood work on the first visit showed white blood cell (WBC) count 2.8 x 10^3^/μL, hematocrit (Hct) 37% and platelet count 96 x 10^3^/μL. There were no significant abnormalities on the peripheral smear and he had a negative direct Coomb’s test. He had a slightly elevated bilirubin, but ferritin, liver transaminases, and vitamin B12 levels were within normal limits. The abnormalities were thought to be secondary to alcohol-related bone marrow suppression, and he was counseled on alcohol cessation and advised to follow up in a month. The repeat lab work one month later showed worsening pancytopenia with his WBCs dropping to 1.6 x 10^3^/μL, Hct to 33% and platelet count to 75 x 10^3^/μL. The physical exam was concerning for splenomegaly which was confirmed by ultrasonography. This raised concern for an underlying hematologic malignancy. A bone marrow biopsy was then performed, and the results were consistent with a trilineage dysplastic process, marked erythroplasia with a few megakaryocytes and blast cells making up less than 5% of all cells. Immunohistochemistry (IHC) revealed 10% of cells to be CD3 and CD5 positive, which raised concern for bone marrow involvement by abnormal T cells. These findings lead to a battery of tests to discern the diagnosis (Table 1).

**Table 1:** Complete blood picture results showing worsening pancytopenia along with results of additional diagnostic lab work ordered. All office visits are roughly one month apart. ALT: alanine aminotransferase, ANA: antinuclear antibody, AST: aspartate aminotransferase, CMV: cytomegaly virus, EBV: Ebstein-Barr virus, Hb: hemoglobin, Hct: hematocrit, LDH: lactate dehydrogenase, MCV: mean corpuscular volume, MDS FISH: myelodysplastic syndrome fluorescence in situ hybridization, PCR: polymerase chain reaction, PNH: paroxysmal nocturnal hemoglobinuria, RBC: red blood cell, RDW: red cell distribution width, WBC: white blood cell. Units: dL: deciliter, g: gram, fL: femtoliter, IU: international units, mg: milligram, mil: million, mL: milliliter, ng: nanogram, μL: microliter.

	Forth Office Visit	Third Office Visit	Second Office Visit	First Office Visit	Reference Range
Complete Blood Picture					
WBC (10^3^/μL)	0.6	1.3	1.6	2.8	4.2-9.1
RBC (mil/Ul)	3.72	3.67	4.01	4.58	4.6-6.1
Hb (g/dL)	9.5	10.2	11.3	12.7	13.7-17.5
Hct (%)	29	30	33	37	40-51
Recticulocytes (%)	5.1	5.1	5.0	4.7	
MCV (fL)	79	82	82	82	79-92
RDW (%)	15.2	15.4	15.3	16.1	11.6-14.4
Platelets (10^3^/μL)	45	63	75	96	150-330
Differential WBC (%)					
Neutrophils	38	53	62	73	
Bands	2	-	-	-	
Lymphocytes	54	44	35	26	
Monocytes	6	2	1	0	
Eosinophils	0	0	0	0	
Basophils	0	0	2	1	
Differential WBC					
Neutrophils (10^3^/μL)	0.2	0.7	1.0	2.0	1.8-5.4
Lymphocytes (10^3^/μL)	0.3	0.6	0.6	0.7	1.3-3.6
Monocytes (10^3^/μL)	0.0	0.0	0.0	0.0	0.3-0.8
Eosinophils (10^3^/μL)	0.0	0.0	0.0	0.0	0.0-0.5
Basophils (10^3^/μL)	0.0	0.0	0.0	0.0	0.0-0.1
Additional tests					
AST (IU/mL)	9	9	12	38	7-37
ALT (IU/mL)	17	18	39	72	10-49
LDH (IU/mL)	153	152	176	146	118-225
Indirect bilirubin (mg/dL)	1.0			1.3	0.1-1.0
Direct bilirubin (mg/dL)	0.5			0.8	0.0-0.3
Haptoglobin (mg/dL)			<1	<1	40-240
Ferritin (ng/dL)				116	22-322
ANA screen			Negative		
EBV PCR			Negative		
CMV PCR			Negative		
PNH immunophenotyping		Negative			
MDS FISH panel		Normal			

**Figure 1:**
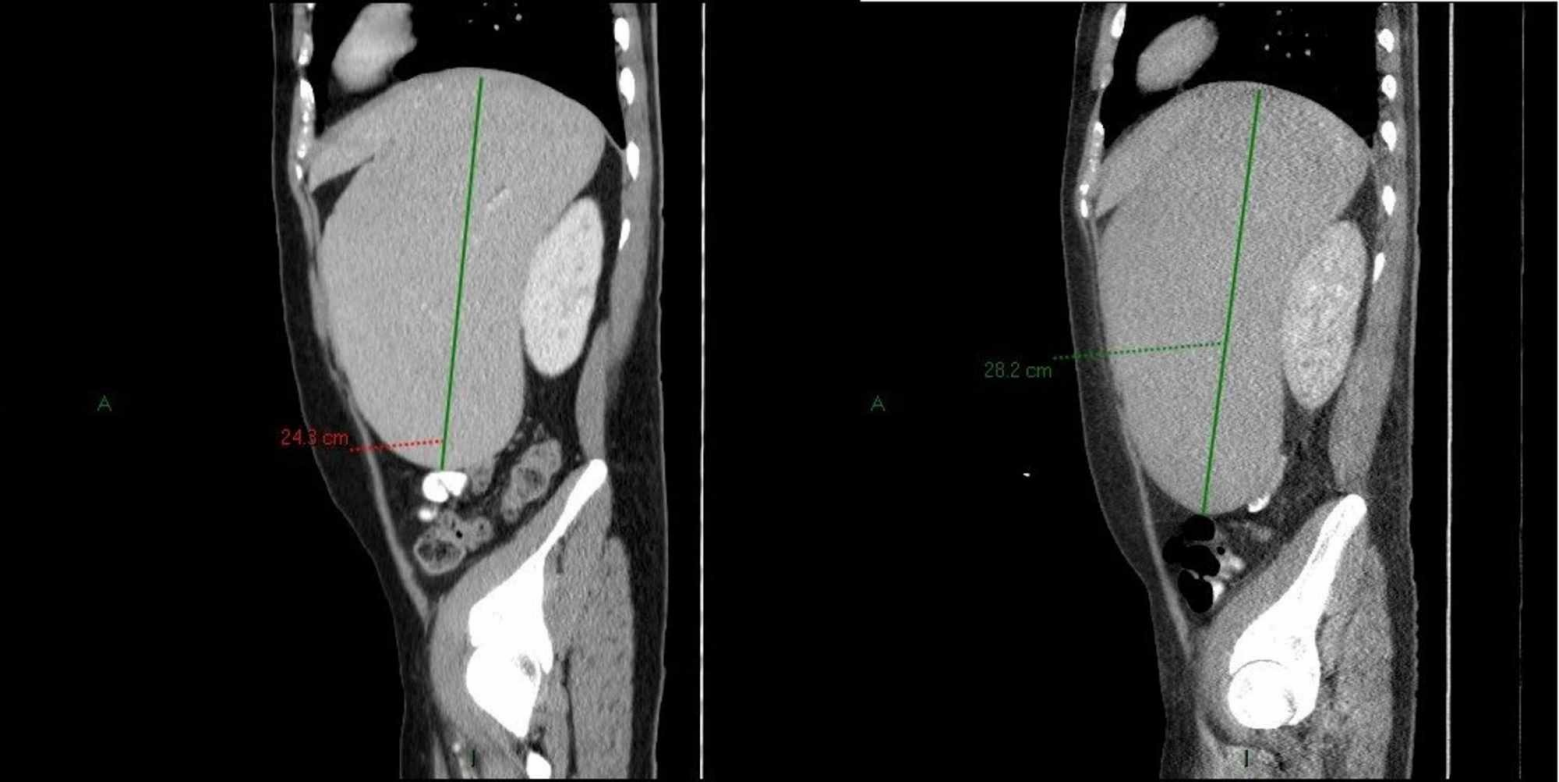
Sagittal section of CT scan showing rapidly progressing splenomegaly with a comparison of two studies done approximately two weeks apart. The spleen can be seen to have enlarged from 24.3 cm (left) to 28.2 cm (right).

Given the dysplastic nature of the marrow cells, myelodysplastic syndrome (MDS) was considered in the initial differential diagnosis but seemed less likely with a negative MDS fluorescence in situ hybridization (FISH) panel. As he had a history of suicide attempts, heavy metal poisoning was considered as a possible cause of early onset MDS but our patient strongly denied any use of heavy metals. Infections like Ebstein-Barr virus (EBV) and cytomegalovirus (CMV) were ruled out with polymerase chain reaction (PCR). Paroxysmal nocturnal hemoglobinuria (PNH) was also considered in light of the negative Coomb’s test and mildly elevated bilirubin in the setting of pancytopenia but the PNH assay was negative. As megakaryocytes were seen in the bone marrow biopsy, idiopathic thrombocytopenic purpura (ITP) was also considered but the steroid trial was deferred because of the atypical presentation and low likelihood. As his blood counts continued to fall, he developed fatigue and exertional dyspnea requiring supportive blood transfusions. His spleen continued to enlarge and in the absence of a definitive diagnosis, a CT scan of the abdomen was done in preparation for a splenectomy for diagnostic purposes, which showed worsening splenomegaly measuring approximately 24 x 20 x 9 cm, for a volume of roughly 4.3 liters. The surgery was delayed as the patient developed neutropenic fever requiring hospitalization. Two weeks later, a repeat CT scan of the abdomen was repeated prior to surgery which showed an increase in the size of the spleen to 28 x 21 x 19 cm (Figure 1).

**Figure 2:**
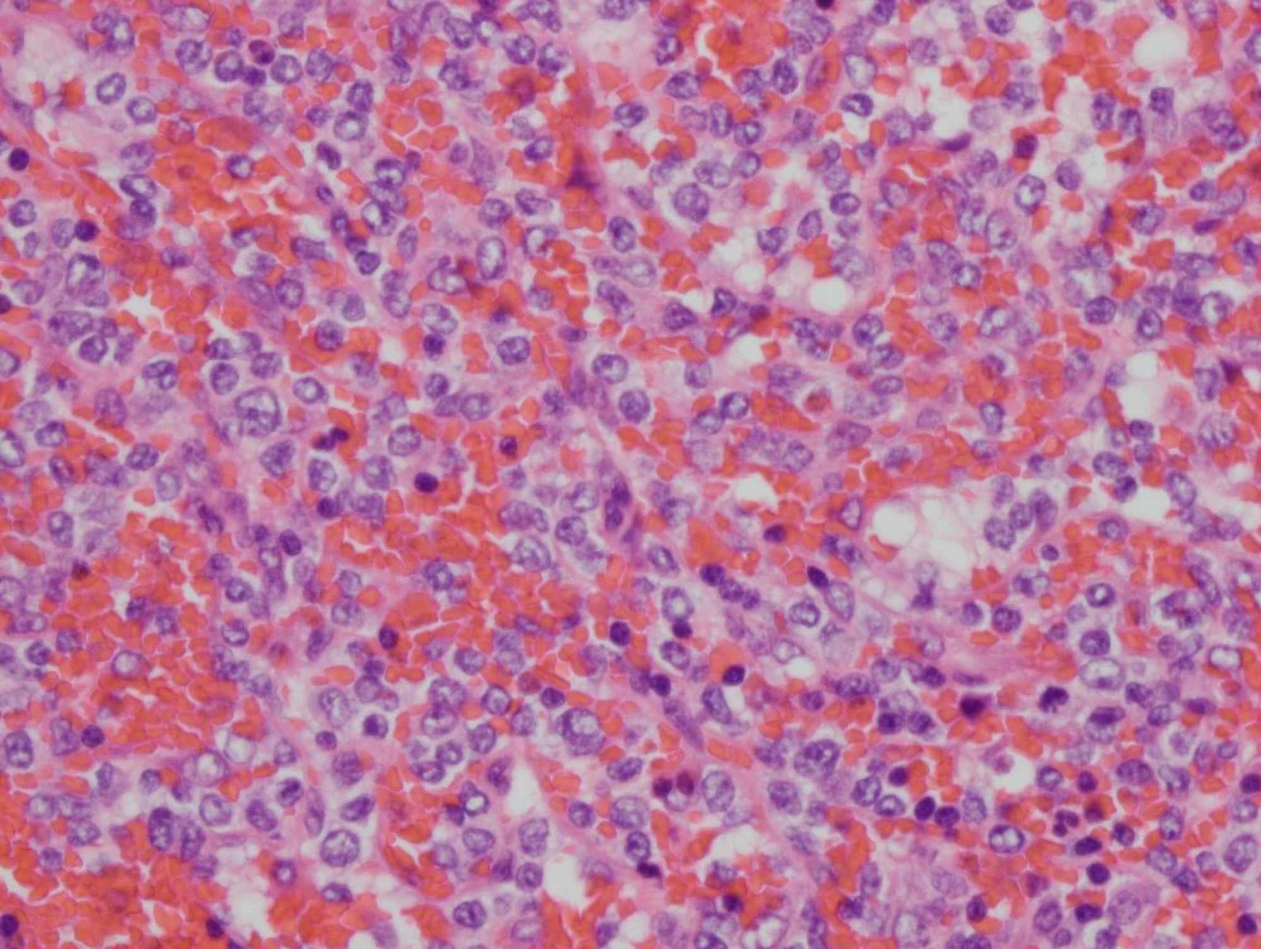
Haemotoxylin and eosin staining of splenic biopsy showing infiltration of the red pulp by atypical lymphoid cells with nuclei containing dense chromatin.

Following splenectomy, the histological examination of the spleen revealed an expanded red pulp infiltrated by a uniform population of medium-sized atypical lymphoid cells (Figure 2).

**Figure 3:**
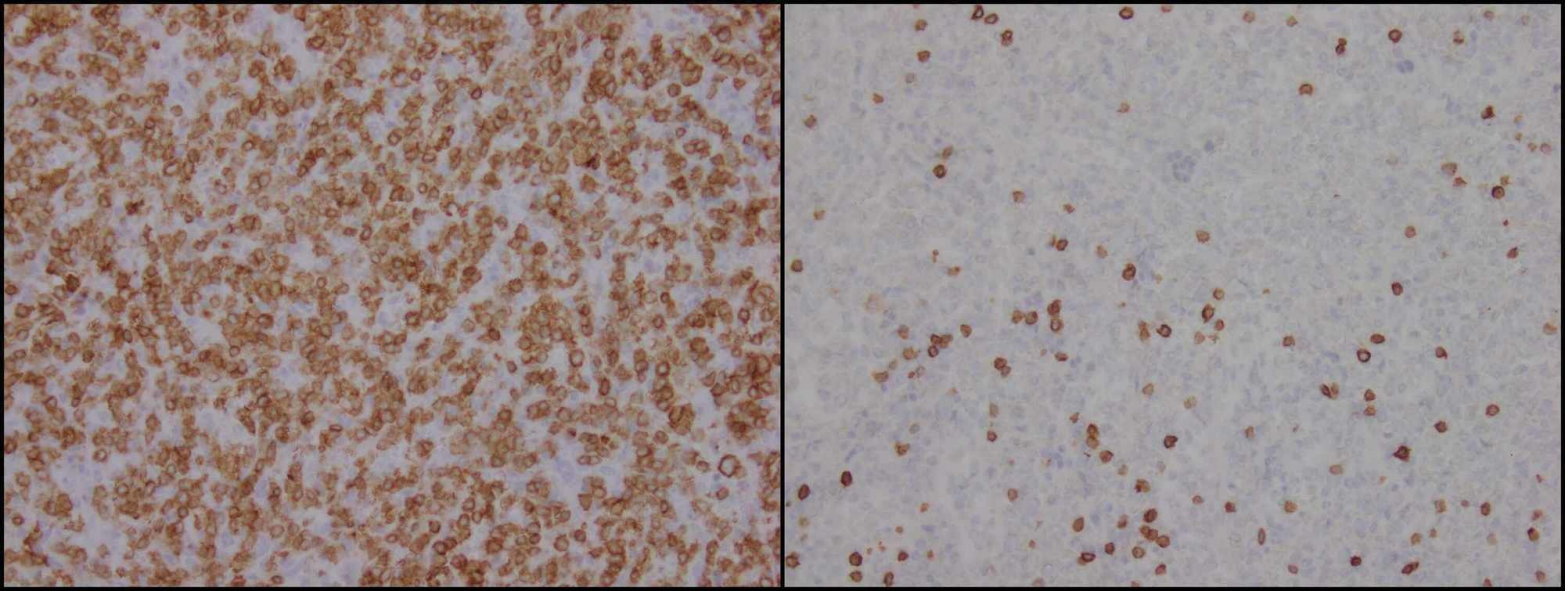
Immunohistochemical staining confirming atypical cells to be T cells which characteristically stain strongly positive for CD3 (right panel) and negative for CD5 (left panel).

**Figure 4:**
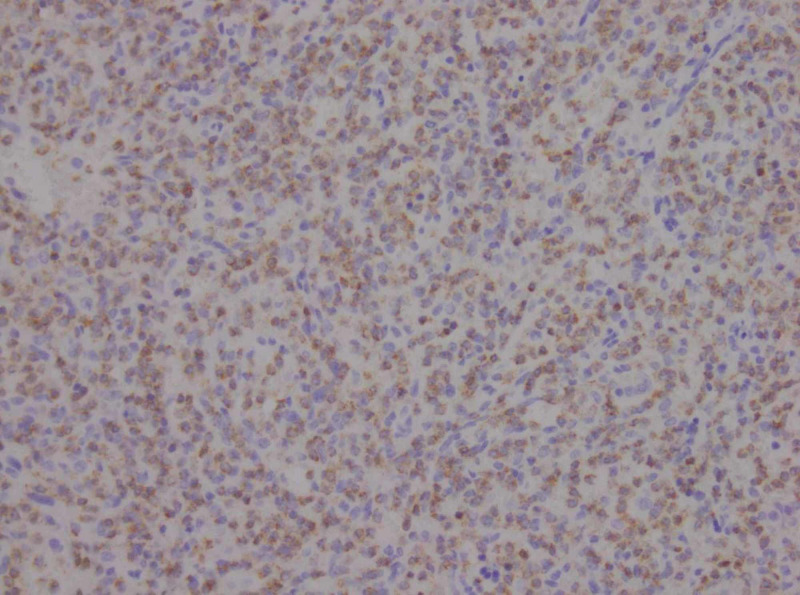
Immunohistochemical staining results showing the atypical T cells to stain positive for T-cell intracytoplasmic antigen (TIA-1) suggestive of hepatosplenic T-cell lymphoma (HSTCL).

IHC revealed these cells to stain positive for CD3 and T-cell intracytoplasmic antigen (TIA-1) (Figures 3, 4). IHC was negative for CD56, CD5 T-cell co-expression and CD20. All these findings were consistent with HSTCL. The recommendations for the patient included a positron emission tomography (PET) scan followed by chemotherapy and an eventual stem cell transplant (SCT); however, he refused all treatment and passed away a few weeks later.

## Discussion

HSTCL is a rare and highly aggressive form of non-Hodgkin's lymphoma that is usually seen in young adults below the age of 40 years and has a five to one male to female predominance [3]. The triad of findings associated with this malignancy consists of pancytopenia, B symptoms and hepatosplenomegaly typically in the absence of lymphadenopathy or peripheral lymphocytosis [3]. Although HSTCL can occur in immunocompetent individuals like our patient, nearly a quarter of the cases are seen in individuals on chronic immunosuppressant therapy like solid organ transplant recipients, and patients receiving treatment for other malignancies or inflammatory bowel disease (IBD) [1-3]. In the subset of patients with IBD, the risk of HSTCL seems to be increased in patients receiving long-term thiopurines as monotherapy or in combination with anti-tumor necrosis factor (TNF) agents [2]. Patients characteristically present with B symptoms like night sweats and fevers along with nonspecific laboratory findings like pancytopenia [2]. These features overlap with several other benign and malignant hematologic diseases which make the diagnosis challenging. The presence of thrombocytopenia with B symptoms and fatigue can be misdiagnosed as mononucleosis in the young age group. The association between EBV and HSTCL has also been explored but no causative link has been established [2]. Similarly, early stages of HSTCL can be confused with protozoal infections like malaria especially in areas where diseases like malaria are endemic [4]. Our patient was relatively asymptomatic and had a history of heavy alcohol use; the initial pancytopenia was regarded to be secondary to ethanol-induced bone marrow suppression. With the elevated bilirubin and anemia, the possibility of PNH was also ruled out. Fanconi’s anemia was also considered because of pancytopenia in the absence of gross bone marrow infiltration. Additionally, malignancy can be confused with ITP in cases where thrombocytopenia improves after splenectomy [4].

As these patients normally do not have lymphadenopathy or abnormalities in the peripheral blood smear, tissue biopsy of bone marrow, liver or spleen is essential to establish a diagnosis that shows infiltration by abnormal T cells in the liver sinusoids and red pulp of the spleen [1]. Flow cytometry of biopsy specimen commonly shows these cells to be positive for CD2, CD3, CD7 and CD26, but negative for CD4, CD5 and CD8. Several molecular cytogenetic abnormalities have also been linked with this malignancy such as an isochromosome of the long arm of chromosome 7 (i(7)(q10) [1,5]. In rare cases, however, malignant cells can be seen in the peripheral blood smear during the leukemic phase of the disease, which can aid in establishing the diagnosis [2].

Although our patient declined treatment, multiple treatment chemotherapy regimens have been tested for induction followed by SCT; however, the median overall survival remains extremely poor with most patients dying because of disease progression within two years of diagnosis [1]. Some literature supports the use of chemotherapy regimens, such as ICE (ifosfamide, carboplatin and etoposide) or IVAC (ifosfamide, etoposide and high-dose cytarabine), rather than the CHOP (cyclophosphamide, doxorubicin hydrochloride, vincristine sulfate and prednisone) regimen, which is often used for aggressive lymphomas. It is also recommended to follow chemotherapy with an allogeneic or autologous SCT; however, data are scarce given the rarity of this condition [2].

## Conclusions

HSTCL is a rare but deadly malignancy. Our case highlights the importance of considering HSTCL in the differential diagnosis of pancytopenia with splenomegaly particularly in young adult males. More studies are needed to determine the optimal therapy in this challenging disease.
